# The Revision of the Materia Medica

**Published:** 1883-10

**Authors:** Ad. Lippe

**Affiliations:** Philadelphia


### THE REVISION OF THE MATERIA MEDICA.

Ad. Lippe, M. D., Philadelphia.

The indefatigable Dr. Richard Hughes published a paper on the revision of the materia medica in the *North American Journal of Homœopathy* for February, 1883, and also in the *British Journal of Homœopathy* for July, 1883. Dr. Hughes first gives the resolutions unanimously adopted by the British Homœopathic Society on March 2d, 1882. The task of reconstructing the materia medica is undertaken by the British Homœopathic Society in view of the considerations as to the state of our materia medica lately adduced by Drs. Yeldham and Black, in England, *and* Dr. J. P. Dake, in America. It is professedly the design of the revisers of our materia medica to expunge all untrustworthy and irrelevant matter. Dr. Hughes admits that the great aim of Hahnemann and his school has accordingly been to ascertain and exhibit the pathogenetic effects of drugs. The object, he says, is beyond all criticism, and the labor and suffering incurred have been above all praise; and had it not been for two unfortunate circumstances the task performed would long ago have compelled the admiration of the profession and might have made homœopathic practice in some measure universal. The features which have ruined it as regards general acceptance and continue to keep it the property of a small minority alone are, he thinks, first, the untrustworthiness of much of its material, and, second, the unintelligible manner of its presentation.

The early practitioners of Homœopathy, as well as a not insignificant number of the practitioners of to-day, have introduced and kept respected the healing art founded by Hahnemann, entirely relying upon the materia medica as they received it from Hahnemann. It is therefore obvious that the present declaration of the untrustworthiness and the unintelligible manner of its presentation has no foundation in fact. The early pioneers and their followers to-day did and do now trust the material left us as an heirloom by Hahnemann; they found its presentation intelligible and its matter trustworthy. The homœopathicians who had learned to utilize the materia medica intelligently not only did not demand a reconstruction, as proposed now by the British Homœopathic Society, but they augmented it and improved the manner of its presentation. Foremost among these men stands the late Dr. Constantine Hering. Dr. Hering did not expunge any untrustworthy and irrelevant matter. His rendition of the pathogenesis of Nux moschata and of Stramonium, above all, his numerous additions to the materia medica, are surely trustworthy and intelligible—certainly intelligible to all homœopathicians who earnestly accept the methods of Hahnemann. They are masterpieces of skill and diligence, and clearly illustrate the possibility of improving, augmenting, and making accessible for curative purposes under the never-failing law of the similars our materia medica. Dr. Hering wrote many papers a long time ago, and also in his later days, about the utter folly of sifting and revising our materia medica. But he did not stop there; he did more for the improvement of our materia medica than any other member of the profession, after Hahnemann, who performed a herculean work when he published *Materia Medica and Chronic Diseases.* And pray, what have Drs. Hughes and Dake done toward the development of our materia medica?

The aim of the revision, we are told in the resolutions, should be to expunge all untrustworthy and irrelevant matter. Later on this ingenious reviser tells us what we shall finally be presented with: "*We shall then have a series of individual pictures of the morbid conditions induced by our medicines, and will only have to fit these to idiopathic disease on the principle similia similibus to have the homœopathic method at our disposal.*" We shall then have "*a pathological picture-book,*" a caricature of a materia medica of which Dr. J. P. Dake has fabled for many years, of which Dr. Hughes is the avowed advocate: Pharmacodynamics first and then the pathological picture-book! Call that revised Homœopathy, revised by men who confess publicly that they do not trust our old-time, honored materia medica because it is "*to them*" unintelligible! These men call for generalization as taught by the common school of medicine: first diagnosticate an idiopathic disease, and then find its similar in the materia medica metamorphosed by Drs. Dake and Hughes into that long-promised pathological picture-book. Labor-saving machines are the order of the day, but as it was in the beginning, so will it be forever—the true healer who fully comprehends the methods of Hahnemann, the founder of our school, must "*individualize.*" We offer correction of the propositions we find here advanced, viz.: *Had it not been for two unfortunate circumstances, the task performed (by Hahnemann, creation of a materia medica) would long ago have compelled the admiration of the profession, and might have made homœopathic practice in some measure universal. The features which have ruined it as regards general acceptance and keep it the property of a small minority alone are:* first, the disloyalty of a not inconsiderable number of professedly homœopathic practitioners to the tenets of our school; second, the declaration on their part that Homœopathy as promulgated by Hahnemann must be "reconstructed" and has been found wanting. It is for this reason that the profession at large does not admire our school. These disloyal pretenders have accepted the name *only*, and we find them both in England and here openly violating one and all of our tenets; we find them demanding recognition by the common school of medicine as a reward for their return to the palliative prevailing allopathic practice, at the same time clamoring for freedom of medical opinion and action, flaunting their disloyalty before the profession, disgracing themselves and trying to disgrace a small minority who have faithfully accepted Hahnemann's teaching and keep it their property. It is not the materia medica of the homœopathic school that wants revision or reconstruction, but the very men who come out with such a preposterous and silly proposition want revision and reconstruction. They are surely not homœopathicians; they are not allopaths, who properly and justly despise them; they are something else—may be they are eclectics to all intents and purposes and should surely join them and be happy.

